# The fluorescent ligand bTVBT2 reveals increased p-tau uptake by retinal microglia in Alzheimer’s disease patients and *App*^NL−F/NL−F^ mice

**DOI:** 10.1186/s13195-023-01375-7

**Published:** 2024-01-02

**Authors:** Cristina Nuñez-Diaz, Emelie Andersson, Nina Schultz, Dovilė Pocevičiūtė, Oskar Hansson, The Netherlands Brain Bank, K Peter R. Nilsson, Malin Wennström

**Affiliations:** 1 Cognitive Disorder Research Unit, Department of Clinical Sciences Malmö, Lund University, Malmö, Sweden; 2 Clinical Memory Research Unit, Department of Clinical Sciences Malmö, Lund University, Malmö, Sweden; 3 Memory Clinic, Skåne University Hospital, Malmö, Sweden; 4 Netherlands Institute for Neuroscience, Meibergdreef 47, 1105 BA Amsterdam, the Netherlands; 5 Department of Physics, Chemistry and Biology IFM, Linköping University, 581 83 Linköping, Sweden

**Keywords:** Retina, Thiophene-based ligands, Alzheimer’s disease, Tauopathy

## Abstract

**Background:**

Amyloid beta (Aβ) deposits and hyperphosphorylated tau (p-tau) accumulation have been identified in the retina of Alzheimer’s disease (AD) patients and transgenic AD mice. Previous studies have shown that retinal microglia engulf Aβ, but this property decreases in AD patients. Whether retinal microglia also take up p-tau and if this event is affected in AD is yet not described. In the current study, we use the p-tau-specific thiophene-based ligand bTVBT2 to investigate the relationship between disease progression and p-tau uptake by microglia in the retina of AD patients and *App*^NL−F/NL−F^ knock-in mice, an AD mouse model known to demonstrate extracellular Aβ plaques and dystrophic neurites in the brain from 6 months of age.

**Methods:**

Evaluation of bTVBT2 specificity and its presence within microglia was assessed by immunofluorescent staining of hippocampal sections and flat-mount retina samples from non-demented controls, AD patients, 3-, 9-, and 12-month-old *App*^NL−F/NL−F^ knock-in mice and 12- and 18-month-old wild type (WT) mice. We used ImageJ to analyze the amount of bTVBT2 inside Iba1-positive microglia. Co-localization between the ligand and p-tau variant Ser396/Ser404 (PHF-1), Aβ, phosphorylated TAR DNA binding protein 43 (pTDP-43), and islet amyloid polypeptide (IAPP) in the brain and retina was analyzed using confocal imaging.

**Results:**

Confocal imaging analysis showed that bTVBT2 binds to PHF-1- and AT8-positive aggregates inside retinal microglia, and not to Aβ, pTDP-43, or IAPP. The density of bTVBT2-positive microglia was higher in cases with a high Aβ load compared to those with a low Aβ load. This density correlated with the neurofibrillary tangle load in the brain, but not with retinal levels of high molecular weight (aggregated) Aβ40 or Aβ42. Analysis of *App*^NL−F/NL−F^ knock-in mouse retina further showed that 50% of microglia in 3-month-old *App*^NL−F/NL−F^ knock-in mice contained bTVBT2. The percentage significantly increased in 9- and 12-month-old mice.

**Conclusion:**

Our study suggests that the microglial capability to uptake p-tau in the retina persists and intensifies with AD progression. These results also highlight bTVBT2 as a ligand of interest in future monitoring of retinal AD pathology.

**Supplementary Information:**

The online version contains supplementary material available at 10.1186/s13195-023-01375-7.

## Background

One of the most characteristic changes in Alzheimer’s disease (AD) is the accumulation of aggregated amyloid beta (Aβ), which deposits in the brain as extracellular plaques. The plaques vary in size and morphology and can be classified into at least 12 different types, of which diffuse and dense-core plaques are the most common [1]. A subset of these dense-core plaques contains dystrophic neurites — abnormal and swollen neuronal processes that contain aggregated hyperphosphorylated tau (p-tau). Such plaques, called cored neuritic Aβ plaques, are closely associated with neuronal loss and cognitive decline in AD [2–4] and the presence of neuritic plaques is today required for neuropathological diagnosis of the disease [4]. Intraneuronal accumulation and aggregation of p-tau are also found in neurofibrillary tangles (NFTs) and neurophil threads (NTs). These structures are spread in a specific pattern throughout the AD brain and can also be observed in other neurodegenerative disorders such as progressive supranuclear palsy (PSP), corticobasal degeneration (CBD), and frontotemporal dementia (FTD) [5]. The Aβ and tau pathology in AD is accompanied by neuroinflammation, seen as activation of astrocytes and microglia. The activated glial cells, in particular activated microglia, are often found encircling neuritic plaques and are currently considered to be an additive hallmark valuable to consider at neuropathological evaluations [1, 6]. Since microglia engulf and degrade both Aβ [7] and p-tau [8, 9], it has been suggested that the function of neuritic plaque-associated microglia is to engulf and degrade the plaque-forming Aβ and p-tau-containing dystrophic neurites.

Interestingly, a growing body of research indicates that the hallmark AD changes, including Aβ deposits [10–13] and p-tau accumulation [10, 13–17], are present in the retinas of both AD patients and transgenic mouse models of the disease. Also, activated microglia [13, 18–20] that have engulfed Aβ have been identified, but the number of microglia engaged in Aβ phagocytosis appears to decrease in patients with mild cognitive impairment and AD compared to controls [13], indicating a reduced capability to remove Aβ as the disease progresses. However, it remains unexplored whether retinal microglia also remove p-tau and how this capability is affected in AD. Notably, these retinal alterations are suggested to occur simultaneously or even before the equivalent changes are detected in the brain [12, 14]. This, in combination with the fact that the retina has the same embryological origin as the brain [21] and is peripherally located (and thereby more accessible compared with the brain), makes the retina highly interesting from a diagnostic perspective. Hence, research on non-invasive imaging methods enabling monitoring of retina’s characteristic AD changes is encouraged and ongoing. In the current study, we aim to determine if a thiophene-based ligand called bTVBT2 can be used to capture AD characteristic changes in the retina. Such thiophene-based ligands produce a conformation-dependent fluorescent illumination when they bind to protein aggregate formations [22]. This property becomes useful in neuropathological evaluation of brain tissue, as these ligands surpass traditional immunostaining in terms of revealing and detecting, for example, Aβ and p-tau aggregates. The ligands are relatively small and peripherally administered ligands have been shown to cross the mouse blood–brain barrier (BBB) and bind to aggregates in vivo [22]. Given the resemblance between the BBB and the blood-retina barrier (BRB) [23], it is plausible that the ligands can also reach and bind to amyloid aggregates in the retina. Thiophene-based ligands may thus be of potential use in the development of future retinal imaging methods. However, although there are numerous commercial and non-commercial thiophene-based ligands available, it is important to point out that their binding is dependent on their molecular structure and can therefore vary in terms of specificity. While, for example, pFTAA, an anionic pentameric oligothiophene, binds to several different types of amyloid aggregates (including Aβ, p-tau, and prions) [22, 24–26], the recently developed ligand bTVBT2 appears to specifically bind to aggregated tau pathologies, and not Aβ, found in human AD brain tissue Sects. [27]. This makes the latter ligand interesting from an AD perspective and could be useful when investigating microglial uptake and degradation of p-tau aggregates in retina. In the current study, we investigated if bTVBT2 binds to structures within microglia in the human retina, if the amount of bTVBT2-containing microglia is altered in AD retina compared to non-demented controls, and if the amount correlates with tauopathy in the brain as well as retinal levels of high molecular weight Aβ (representing levels of highly aggregated Aβ). We also investigated if the presence of bTVBT2-bound structures in microglia is related to disease progression by analyzing retinae from the AD knock-in mouse model *App*^NL−F/NL−F^ at ages before and after the formation of Aβ plaques and dystrophic neurites.

## Methods

### Individuals included in the study

Retina samples from non-demented controls (*n* = 8) and clinically diagnosed AD cases (*n* = 9) as well as hippocampal samples from clinically diagnosed AD cases (*n* = 4) acquired through a collaboration with The Netherlands Brain Bank (NBB) were included in the study. None of the cases had a history of ophthalmological disease or injuries. All donors provided informed consent for retina and brain autopsies and for the use of material and clinical data for research purposes in compliance with national ethical guidelines. Clinical diagnoses, sex, age, neuropathological assessment (neurofibrillary tangles (NFT) scored according to Braak stages I-VI [28] and Aβ plaques scored into O, A, B, C, where O = zero, A = some, B = moderate, and C = many [28]), *APOE* genotype and cause of death are presented in Tables 1 and 2. Levels of high molecular weight Aβ40 and Aβ42 in (*n* = 14) of the cases were measured previously [29] using MesoScale Discovery V-plex A Peptide Panel 1 kit with electrochemiluminescence detection technology (MesoScale Discovery, Rockville, MD) according to manufacturers’ protocol. The Aβ40 and Aβ42 levels are presented in Table 1.

**Table 1:** Clinical diagnosis, sex, age, neuropathological assessment, bTVBT2 + microglia density and cause of death of cases included in the retina study

Clinical diagnosis	Sex	Age (years)	Braak stages (NFT/Aβ/LB)	APOE genotype	PMD (h:min)	Aβ40_HMW_ (pg/ml)	Aβ42_HMW_ (pg/ml)	Cause of death
NC	M	81	3/C/0	4/3	4:30	23.48	1.83	Pancreas carcinoma
NC	F	92	3/O/1	3/4	6:35	2.80	0.45	Heart failure
NC	M	75	1/A/0	3/3	7:10	29.33	4.31	Cardiac arrest
NC	M	70	1/O/3	3/2	6:20	2.85	0.19	Pneumonia
NC	M	102	3/A/0	4/3	5:00	24.16	3.09	Ileus
NC	M	55	0/O/0	3/3	7:30	12.10	0.21	Esophageal cancer
NC	F	60	0/O/0	3/2	8:10	0.67	0.07	Breast cancer
NC	F	68	1/O/0	3/3	4:30	na	na	Euthanasia
AD	F	92	6/C/0	4/3	6:10	4.91	0.92	Atrioventricular block
AD	F	70	6/C/0	4/4	4:20	8.12	1.33	Cachexia
AD	F	78	6/C/0	4/4	4:45	18.36	2.26	Cachexia
AD	F	96	4/B/0	3/3	7:20	4.78	0.34	Heart failure
AD	M	69	6/C/0	4/3	6:30	na	na	Pneumonia
AD	F	65	5/C/0	4/3	4:30	na	na	Respiratory infection
AD	F	88	5/C/5	3/3	5:40	2.28	0.27	Cachexia
AD	M	63	4/C/6	4/4	4:55	3.03	0.40	Hepatic insufficiency
AD	F	83	4/C/6	3/3	6:15	1.81	.018	Respiratory infection

Cases clinically diagnosed as non-demented controls (NC) or Alzheimer’s disease (AD). Braak staging of neurofibrillary tangles (NFT), amyloid (Aβ), and Lewy bodies (LB). *F *Female, *M *Male. Aβ40_HMW_ = high molecular weight amyloid beta 40. Aβ42_HMW_ = high molecular weight amyloid beta 42. na = not analyzed due to lack of tissue

**Table 2:** Clinical diagnosis, sex, age, neuropathological assessment, and cause of death of cases included in the hippocampal study

Clinical diagnosis	Sex	Age (years)	Braak stages (NFT/Aβ/LB)	APOE genotype	PMD (h:min)	Cause of death
AD	M	68	5/C/0	3/3	9:15	Euthanasia
AD	F	81	4/C/0	4/3	8:10	Pyelonefritis
AD	F	84	4/C/0	4/4	7:32	Viral infection
AD	M	72	6/C/0	4/3	5:15	Pneumonia and stomach bleeding

Individuals clinically diagnosed as patients with Alzheimer’s disease (AD), included in the study. Braak staging of neurofibrillary tangles (NFT) and Lewy bodies (LB) and ABC staging of amyloid (Aβ). *F *Female, *M *Male

### Human retina and hippocampal samples

**Fig. 1:**
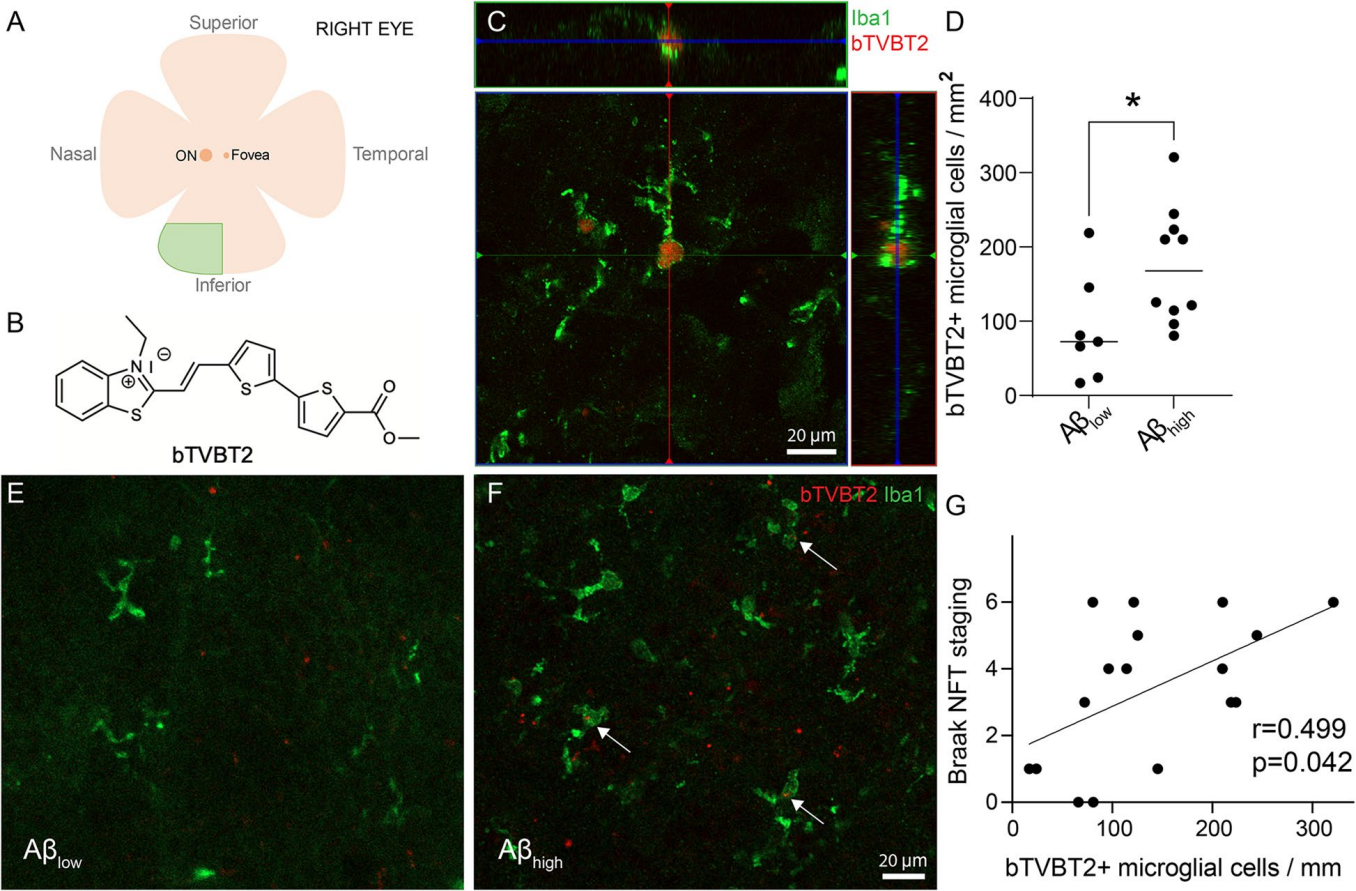
bTVBT2 inside microglia in the human retina. A representation of the human retina showing the region used in this study (inferior-nasal, green field) is seen in **A**. The molecular structure of bTVBT2 is shown in **B**. Confocal image of the orthogonal view of a bTVBT2-positive signal inside an Iba1-positive microglia is shown in **C**. Graph in **D** shows a higher number of bTVBT2-positive microglia density in the retina of Aβ_high_ cases compared to Aβ_low_ cases. Data was analyzed using the Mann–Whitney *U* test and is presented as a median with a 95% confidence interval. Significant difference at * = *p* < 0.05. A case with low amyloid-beta load (Aβ_low_) showing no bTVBT2 inside retinal microglia is seen in **E**, and a case with high amyloid-beta load (Aβ_high_) with bTVBT2-positive deposits inside retinal microglia (white arrows) is shown in **F**. Scale bar in **B** represent 20 µm and scale bar in **D** and **E** represent 20 µm. Scatter plot in **G** shows the correlation between bTVBT2-positive microglia density in the retina and NFT staging in the brain. Data was analyzed using Spearman’s rank correlation coefficient

The right eyeballs of the controls and AD cases were enucleated, and the lens, cornea, and vitreous humor were removed. The eyecups were thereafter filled with Tissue-Tek O.C.T. Compound (Sakura Finetek, Torrance, CA), snap frozen with isopentane cooled with liquid nitrogen, and kept at − 80 °C until used. The eyecups were divided into four segments, and the peripheral inferior-nasal part (see Fig. 1A) was dissected and fixed in 4% paraformaldehyde for 4 h. During this incubation the retina detaches from the sclera and after being washed in PBS, the retina was kept in an antifreeze solution (30% ethylene glycol and 30% glycerol in 0.5 M PBS) at − 20 °C. The human hippocampal tissue was immersion-fixed in 4% paraformaldehyde and left in phosphate-buffered saline with 30% sucrose for 3 days. Thereafter the tissue was sectioned with a microtome (Leica SM 2010R) into 40 µm-thick sections and kept free-floating in an antifreeze solution at − 20 °C.

### Mice retina samples

The *App*^NL−F/NL−F^ knock-in mice express humanized Aβ with the Swedish (APP K670N/M671L) and Iberian (APP I716F) mutations, under the endogenous mouse amyloid precursor protein (APP) promoter. Depositions of Aβ and dystrophic neurites start to develop at 6 months of age in the cortex and hippocampus, while NFT and NT are not formed [30]. In this experiment, the *App*^NL−F/NL−F^ knock-in mice and wild type (WT) littermates were housed in groups of 2–5 mice per cage under a 12:12-h light/dark cycle with food and water provided ad libitum. The *App*^NL−F/NL−F^ knock-in mice were sacrificed at 3 months (*n* = 6, *n* = 4 females and *n* = 2 male) and 9–12 months (*n* = 6 females) of age, while WT littermates were sacrificed at 12 and 18 months of age (*n* = 4 males, *n* = 2 females). The eyes were collected following transcardial perfusion with ice-cold 0.1 M phosphate buffer by removing the entire ocular globe after breaking the maxillary bone. A needle was used to puncture the cornea, and the whole ocular globes were fixed in 4% paraformaldehyde for 4 h and thereafter left in 0.1 M phosphate buffer at 4 °C for 1 day. To extract the retina, a Vannas scissors were used to create a circular incision around the cornea, separating the posterior and anterior segments of the eye. Gentle pressure was then applied to the posterior part of the sclera to remove the lens and vitreous. The neural retina is loosely attached to the retinal pigment epithelium (RPE), so it was dissected by gently squeezing the eye from posterior to anterior, pulling the tissue that was then collected by using a fine forceps and thereafter kept in antifreeze solution at − 20 °C.

### Immunostaining

The brain sections and the flat-mount retina samples were first washed with KPBS, followed by incubation in blocking solution, consisting of 5% normal goat serum (Millipore, S26-100ML) in KPBS Triton X-100 0.25%, for 1 h at room temperature. Then, the tissue was incubated in primary antibody (antibody targets, catalog names, isotypes, concentrations, and vendors are listed in Table 3) in a blocking solution for 72 h at 4 °C. Afterwards, the tissue was washed with KPBS Triton X-100 0.25% and incubated in the appropriate secondary antibody (Goat anti-rabbit 488 1:200 (Invitrogen, A11008), goat anti-mouse 488 1:200 (Invitrogen, A11029) and/or goat anti-rabbit Cy5 1:200 (Invitrogen, A10523)) in blocking solution for 24 h at 4 °C. After washing with KPBS, the tissue was stained with the ligand bTVBT2 which was diluted 1:2000 in PBS from a stock solution (1.5 mM in DMSO), for 1 h at room temperature. Thereafter the sections were incubated in Sudan Black (1% in 70% ethanol) (Sigma-Aldrich) for 5 min before they were mounted with Vectashield mounting medium containing DAPI (Vector Laboratories). Hippocampal sections and retina flat-mount samples stained according to the above protocol, but with KBPS instead of the primary antibodies and/or bTVBT2, were used as negative controls. Sections of the hippocampus were used as positive controls when retina samples were stained for bTVBT2/Iba1/PHF1.

**Table 3:** Antibodies used in the study

Target	Antibody	Isotype	Concentration	Source
p-tau181	AT270	Mouse IgG	1:200	Thermo Fisher Scientific
p-tau202/205	AT8	Mouse IgG	1:200	Thermo Fisher Scientific
p-tau369/404	PHF1	Mouse IgG	1:200	Dr Peter Davies
IAPP	T-4149	Rabbit IgG	1:200	Peninsula
Tau	A0024	Rabbit IgG	1:200	Dako
p-TDP-43	22,309–1-AP	Rabbit IgG	1:200	Proteintech
Aβ 40/42	MOAB-2	Mouse IgG	1:200	Biosensis,
Aβ 1–16	6E10	mouse IgG	1:200	Biolegend
Iba-1	019–19741	Rabbit IgG	1:200	Wako

### Imaging and quantifications

The presence of bTVBT2 inside microglia located between the outer plexiform (OPL) and the ganlion cell layer (GCL) in both human and mouse retinas was analyzed using confocal imaging (Zeiss LSM 510) of Iba1/bTVBT2-stained tissue. The bTVBT2 signal within microglia was confirmed using Lambda Scan imaging (Zeiss LSM 510). The emission spectra of the areas positive for bTVBT2 inside Iba1-positive microglia were measured and compared with the known spectrum of bTVBT2 (with an emission maximum of around 600 nm), as described previously [27]. The percentage of bTVBT2-containing microglia was manually quantified by randomly selecting (*n* = 50 per individual) Iba-1 positive cells with characteristic microglial appearances (cell bodies with processes) in the green filter (the Iba1 staining). Confocal images of the microglia were thereafter captured the green (Iba1) and red channel (bTVBT2) using the 63 × objective. The presence of bTVBT2 inside microglia was confirmed by acquiring Z-stacks (spaced 1 µm apart) and microglia demonstrating bTVBT2 encapsulated by Iba1 staining was considered as a bTVBT2 containing microglia. The percentage of microglia with bTVBT2 as well as bTVBT2-positive microglia per microglia density was calculated and presented as bTVBT2 + microglial cells/mm^2^. Co-localization of bTVBT2 with different tau markers in dystrophic neurites was analyzed by capturing pictures of 10 clustered dystrophic neurites in Cornu ammonis 1 (CA1) of (*n* = 3) AD cases (in total 30 clustered dystrophic neurites in each staining) using an Olympus BX41 light microscope with 40 × objective. Using ImageJ software, a threshold was set for the detection of bTVBT2 (red channel) and the different tau markers (green channel). The bTVBT2-positive area, as well as the area of colocalization with tau, were measured for each dystrophic neurite cluster. The percentage of the total bTVBT2-positive area that co-localized with tau was calculated for each marker (tau, p-tau181, AT8, and PHF-1).

### Statistics

The Prism software (version 9.2.0, GraphPad) was used for statistical analysis and graphical representation. The Kolmogorov–Smirnov test was performed for assessing normal distribution. For normally distributed data, a one-way ANOVA followed by the Tukey test was used. For non-normally distributed data, the Mann–Whitney *U* test was performed. Normally distributed data are represented as means ± standard deviations, while non-normally distributed data are represented as medians. Individual values are shown in both cases. For correlation assessment, Spearman’s rank correlation coefficient was used, and data are represented as scatter plots. A value of *p* < 0.05 was considered significant.

## Results

### Cases with high Aβ load show a higher amount of bTVBT2-containing microglia in the retina

Analysis of Iba1/bTVBT2-stained retina samples from the inferior-nasal peripheral part (Fig. 1A) showed that bTVBT2 (molecular structure seen in Fig. 1B) was found in the retinas of both controls and AD cases (Fig. 1C). The number of bTVBT2-containing microglia was not significantly increased in AD retinas when comparing control cases with AD cases (76.63 vs 125.40, respectively, *p* = 0.139). However, since the neuropathological evaluation revealed that one control case demonstrated a high Aβ score (i.e., C), we also stratified the cohort according to Braak staging into low (O-A) vs high (B-C) brain Aβ load. Analysis after this stratification showed that the number of bTVBT2-containing microglia was significantly higher in cases with a high Aβ load (Fig. 1D–F). Furthermore, bTVBT2-positive microglia density correlated significantly with brain Braak NFT scores (Fig. 1G), but we found no correlation with retinal levels of high molecular weight Aβ40 or Aβ42 (*r* = 0.059, *p* = 0.840 and *r* = 0.064, *p* = 0.829, respectively) (Supplementary Fig. 1A and B).

### bTVBT2 in retinal microglia co-localizes with PHF-1 and AT8 immunoreactivity

**Fig. 2:**
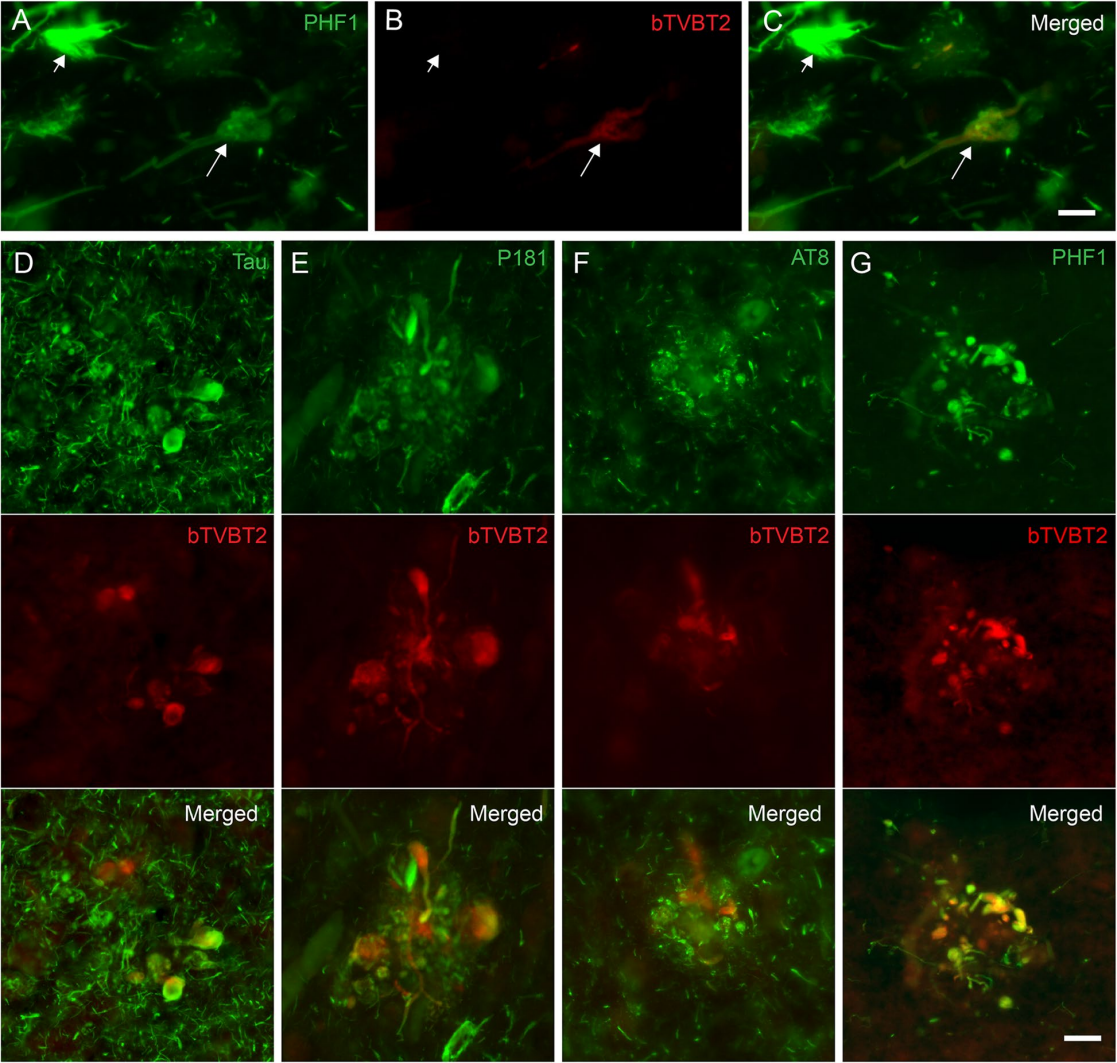
bTVBT2 co-localization with tau in human hippocampus. Cornu ammonis 1 (CA1) from an AD case stained against tau (**A**), bTVBT2 (**B**), and merged image (**C**), showing little (long arrows) or no (short arrows) co-localization of bTVBT2 with PHF-1-positive NFTs. Hippocampal sections stained with bTVBT2 together with tau (**D**), p-tau181 (**E**), AT8 (**F**), and PHF-1 (**G**) showing co-localization of bTVBT2 with tau-positive dystrophic neurites. Scale bars in **A**–**C** and **D**–**G** represent 10 µm and 20 µm, respectively

**Fig. 3:**
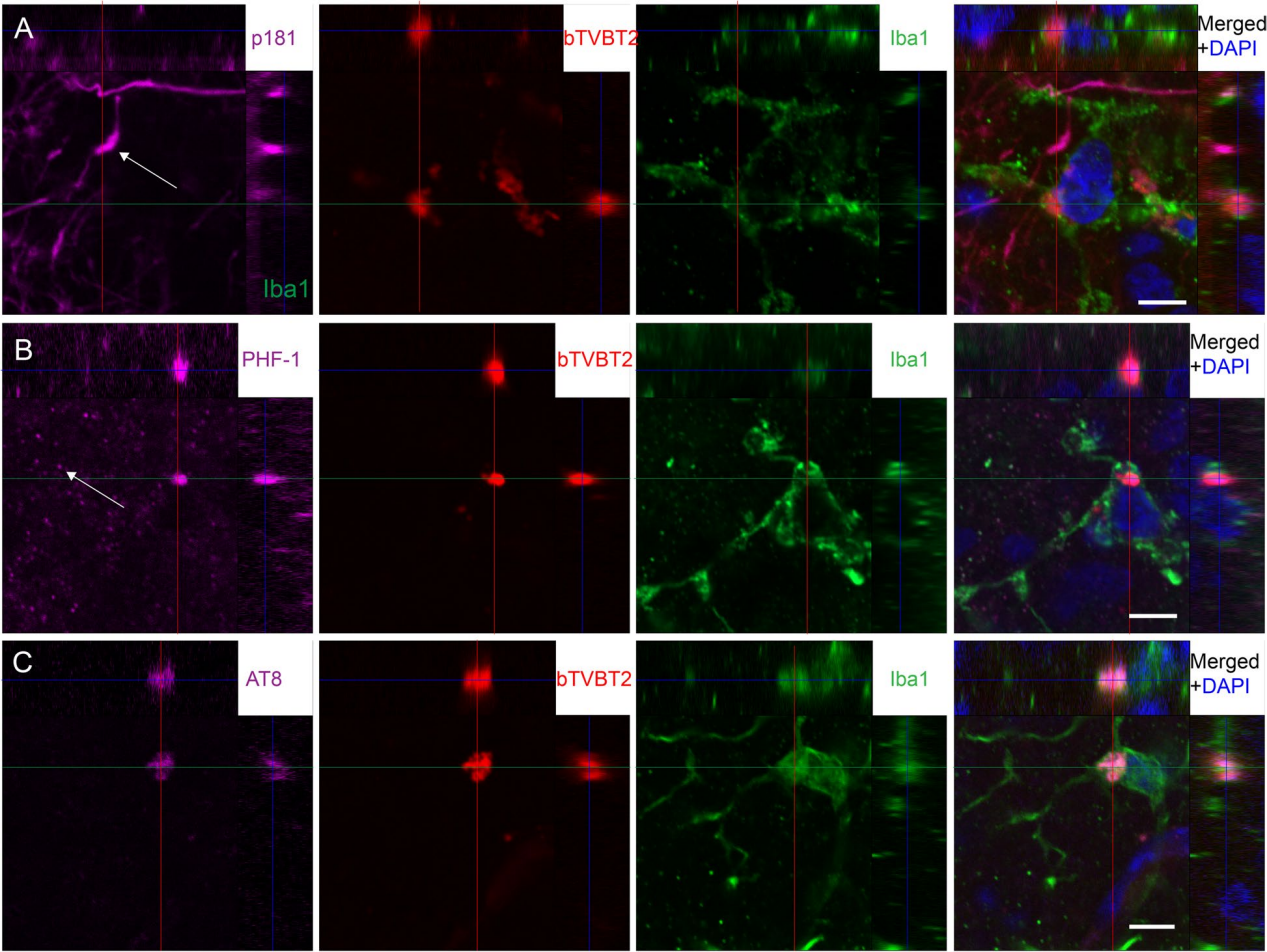
bTVBT2 co-localization with AT8 and PHF-1 but not p-tau181 in human retina. Confocal image of the orthogonal view of a flat-mount retina sample from an AD stained against p-tau181 (**A**) (purple indicated with an arrow), PHF-1 (**B**) (purple indicated with arrow), and AT8 (**C**) (purple) together with bTVBT2 (red in **A**–**C**) and Iba-1 (green in **A**–**C**). The three p-tau stainings are merged with a staining against DAPI (purple, red, green, and blue in **A**–**C**). Scale bars in (**A**–**C**), represent 5 µm

To further identify the aggregates taken up by the retinal microglia, and to spare the unique and less accessible retinal tissue, we initiated our evaluation by double staining hippocampal samples against bTVBT2 together with antibodies directed against all forms of Aβ42 (i.e., unaggregated, oligomers and fibrils) and unaggregated Aβ40, phosphorylated TAR DNA-binding protein-43 (pTDP-43), islet amyloid polypeptide (IAPP), tau, p-tau181, AT8 and PHF-1. In accordance with previous studies [27], the staining showed that bTVBT2 in the CA1 region co-localized with a few NFTs and NTs (Fig. 2A–C) but was found foremost co-localized with dystrophic neurites (Fig. 2D–G). The co-localization in dystrophic neurites differed between tau/p-tau variants, and while PHF-1 showed a co-localization with a median of 57% of the bTVBT2, only 30%, 16%, and 42% of p-tau181, AT8 and tau, respectively, co-localized with bTVBT2 (Fig. 2D–G). No co-localization was detected between bTVBT2 and Aβ, pTDP-43 or IAPP in the hippocampus of any of the cases (see Supplementary Fig. 2). Next, retina samples from AD cases were stained against p-tau181, PHF-1, and AT8 together with Iba-1 and bTVBT2. The p-tau181 staining revealed strong immunoreactive processes (indicated with an arrow in Fig. 3A), while the PHF-1 immunoreactivity instead was seen as a dotted pattern (indicated with an arrow in Fig. 3B). The staining using AT8, showed an overall very little immunoreactivity (Fig. 3C). Both PHF-1 and AT8 immunoreactivity, but very little or no p-tau181 immunoreactivity co-localized with the bTVBT2 (Fig. 3A–C). None of the bTVBT2 within retinal microglia co-localized with Aβ, pTDP-43 or IAPP.

### Proportion bTVBT2-containing retinal microglia in App^NL−F/NL−F^ knock-in mice^F^ increases with disease progression

**Fig. 4:**
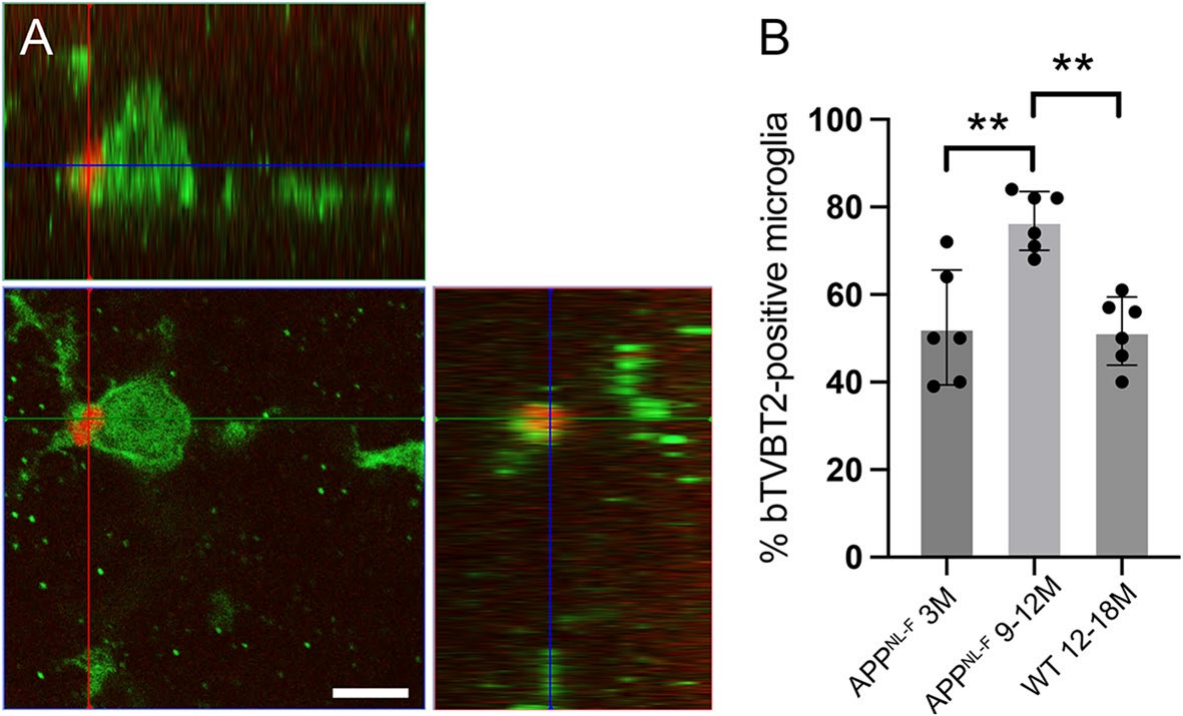
bTVBT2 co-localization with tau in mouse retina. Image in **A** shows a confocal orthogonal view of a bTVBT2-positive signal inside an Iba1-positive retinal microglia in a 3-month-old *App*^NL−F/NL−F^ knock-in mouse. The graph in **B** shows that the proportion of bTVBT2-positive microglia in the retina increases significantly in 9–12-month-old *App*^NL−F/NL−F^ knock-in mice compared to 3-month-old *App*^NL−F/NL−F^ knock-in mice, whereas 12- to 18-month-old WT mice have similar levels as 3-month-old *App*^NL−F/NL−F^ knock-in mice. Data is presented as mean ± SD and was analyzed using one-way ANOVA, followed by the Tukey test with (*n* = 3) comparisons. Each point represents the mean of (*n* = 50) microglia in each group (*n* = 6). Significant difference at ** = *p* < 0.01. Scale bar in **A** represents 5 µm

Next, we investigated if the uptake of bTVBT2 by retinal microglia is associated with AD pathology progression by analyzing bTVBT2-containing microglia in the retina of 3-month-old (before AD pathology onset) and 9–12-month-old (after AD pathology onset) *App*^NL−F/NL−F^ knock-in mice and compared it with 12–18 month-old WT mice. As in humans, *App*^NL−F/NL−F^ knock-in mice showed bTVBT2 structures within retinal microglia (Fig. 4A). Interestingly, the presence of bTVBT2-containing microglia in *App*^NL−F/NL−F^ knock-in mice was rather high already at 3 months (approximately 50% of the analyzed microglia), still not differing significantly from the presence in 12–18-month-old WT mice. The amount of bTVBT2-containing retinal microglia was significantly higher in 9–12-month-old *App*^NL−F/NL−F^ knock-in mice compared to 3-month-old *App*^NL−F/NL−F^ knock-in mice and compared to 12–18-month-old WT mice (Fig. 4B).

## Discussion

In the current study, we used bTVBT2 to detect p-tau aggregates inside retinal microglia in AD patients and *App*^NL−F/NL−F^ knock-in mice. The specificity of the ligand was evaluated by staining AD hippocampal samples which showed that, consistent with previous studies [27], bTVBT2 binds to aggregated tau pathologies such as NTs and NFTs, but it is most pronounced in dystrophic neurites. It is interesting to note that staining against different p-tau variants yielded varying percentages of overlap, where the PHF-1 staining gave rise to the highest percentage. This result may indicate that the bTVBT2 binds aggregates in the dystrophic neurites to a higher degree formed by tau phosphorylated at Ser396/404 compared to tau phosphorylated at other sites we examined. Importantly, dystrophic neurites also contain other proteins including the APP and lysosomal proteins [31]. Thus, although we found no overlap between the ligand and Aβ, IAPP, and pTDP-43, we cannot entirely exclude the possibility that bTVBT2 also binds to other amyloid protein aggregates within the dystrophic neurites. Nevertheless, and most importantly for our study, the bTVBT2 inside the AD retinal microglia was positive for both PHF-1 and AT8, two well-known markers for tauopathy in the brain. Surprisingly, no co-localization was found between retinal p-tau181 and bTVBT2. However, although this marker also detects brain tauopathy, recent studies suggest that p-tau181 has a physiological role in the retina [17]. Indeed, our p-tau181 staining yielded a robust immunoreactivity pattern resembling neuronal processes, which supports this idea. These p-tau181 results stand in contrast to the AT8 staining (which showed binding to tau phosphorylated at Ser202 and Thr205), which yielded much less immunoreactivity and were foremost found as aggregates co-localizing with bTVBT2. Hence, AT8 appears to be a more reliant marker for AD-specific tauopathy in the retina, which also has been suggested in recent studies [17].

We further found no co-localization between bTVBT2 and Aβ, IAPP, or pTDP-43, and the percentage of bTVBT2-containing microglia, and the retinal levels of high molecular weight Aβ (theoretically corresponding to aggregated Aβ) did not correlate. Hence, although uptake of Aβ by retinal microglia has been described [13], we conclude that the bTVBT2-positive aggregates taken up by microglia are most likely formed by tau and not by Aβ. This conclusion finds support in previous in vitro and in vivo mouse studies that have shown the internalization of soluble and insoluble tau by microglia [8, 9].

The bTVBT2-positive microglia density in cases with high Aβ load was higher compared to cases with low Aβ load. This finding could be a result of the previously shown increase in p-tau (and thereby the increased need of removal) in the AD retina [10, 13–16]. This increase in p-tau uptake contrasts with the previous study demonstrating reduced microglial Aβ uptake in the AD retina [13], but while the latter is suggested to be due to a reduced capacity to remove Aβ (and thereby contributing to the accumulation and deposition of Aβ in brain and retina), microglial p-tau engulfment may contribute to AD pathology in a different manner. While microglia take up p-tau to degrade it [9], they also re-release p-tau in exosomes [32, 33]. Such exosomes have been found to a greater extent in cerebrospinal fluid and blood of AD patients [33, 34] and it has therefore been proposed that microglia help to propagate tauopathy by engulfing, re-packaging, and releasing tau aggregates [35]. Support for this idea was found in a study demonstrating the impediment of tauopathy after depleting microglia or inhibiting tau microglial release in tauopathy mouse models [32]. Therefore, it may be that the enhanced density of bTVBT2-containing microglia seen in our study contributes to the increased presence of p-tau in the AD retina. It should however be noted that the staining pattern of retinal p-tau in previous studies is rather dispersed and structures such as NTs and NFTs have not been reported [17]. Our bTVBT2 staining also did not capture classical NT or NFT structures, and hence, we speculate that the aggregation of p-tau occurs once it is taken up by microglia.

The increase in density of bTVBT2-containing microglia correlated significantly with Braak NFT stages, indicating that this event co-occurs with enhanced tauopathy in the brain. The correlation was, however, only modest and the density of bTVBT2-containing microglia was not higher in controls (NFT Braak stage 3 and below) compared to AD patients (NFT Braak stage 4 and above). This could be because NFT Braak stages represent stages of NFT spreading, while bTVBT2 foremost captures aggregates in dystrophic neurites (which are foremost found in Aβ plaques). This would explain why a difference in the density of bTVBT2-containing microglia only was detected when the cohort was stratified on Aβ scores. From this perspective, it would be of interest to investigate a potential correlation with a neuropathological evaluation according to CERAD, which scores neuritic plaques throughout the brain [4]. Unfortunately, the collection of retinas was performed before this scoring system was implemented at the NBB and hence, we are unable to investigate such a correlation.

When analyzing the retina of *App*^NL−F/NL−F^ knock-in mice, we noted that as many as approximately 50% of the retinal microglia contained bTVBT2 at 3 months of age. At this age, neither Aβ plaques nor dystrophic neurites have been formed [30], and since a similar percentage was found in 12- to 18-month-old WT mice, we speculate that the uptake and degradation of tau in mice retina is an ongoing process unrelated to AD pathology. The increase of bTVBT2-containing microglia in 9- and 12-month-old *App*^NL−F/NL−F^ knock-in mice may reflect, in similarity to the human AD retina, an augmented AD pathology, which in the brain is evident from 6 months of age in these mice.

Besides the limitations mentioned above, there are additional important limitations to point out. One of these limitations is the case-cohort size. Very few brain banks collect and store postmortem eyes in the manner required to perform the flat-mount staining and the most common way to analyze retinas involves cross-sectioning. This method gives many times more sections to analyze, but in turn, it makes it more difficult to retrieve important information such as structural differences and morphological changes. We therefore would like to encourage the collection and storage of retinas in both ways to enable further studies on larger cohorts. Another limitation is the method of retina sample collection. Previous studies on retinal p-tau and Aβ aggregates in AD patients have mostly been conducted on samples from either flat-mount entire human retinas or cross-sections of the nasal-temporal part of the retina [11, 13, 17]. In contrast, our study was performed on flat-mount samples of a part of the inferior nasal retina. Since previous studies suggest that AD pathology may differ between regions of the retina [15], we cannot exclude the possibility that microglia in other parts of the retina may exhibit a different aggregate pattern. Finally, there are several different transgenic AD mouse models available today, but each model mimics only certain aspects of the AD pathology seen in humans. Hence, when choosing a model for our experiments, we needed to consider both the relevance and as well as the strengths and weaknesses of different models. Since the purpose of our transgenic AD mouse study was to investigate if the microglial uptake of p-tau is associated with AD pathology, we needed a model with a slow AD pathology progression. This is to ensure that we could collect fully matured retinas both before and after visible signs of AD pathology. We also needed a model that demonstrates dystrophic neurites, the p-tau structures we know the ligand binds to. Our model of choice, the *App*^NL−F/NL−F^ knock-in mice, shows both Aβ plaques and dystrophic neurites at 6 months of age, which successively and regardless of sex [36] increases over time much like the AD pathology progression seen in humans. Hence, the model fulfills the two most important criteria for our study. However, the *App*^NL−F/NL−F^ knock-in mice lack other AD characteristic pathologies including tau tangles (although different p-tau variants are present in both the brain and retina). In addition, only the 3-month-old *App*^NL−F/NL−F^ knock-in mice group and WT group contained males. Although sex differences in regard of AD pathology have not previously been shown in *App*^NL−F/NL−F^ knock-in mice [36], studies have shown that microglia density, activation, and antigen presentation (but not phagocytosis) in the brain differs between female and male mice [37]. We can therefore not exclude the possibility that microglial uptake in the retina also is affected by sex. Hence, our results should be interpreted with care and viewed foremost as results supporting the idea that microglia p-tau uptake is increased along with AD pathology.

## Conclusion

Our study shows that p-tau aggregates can be found within the retinal microglia in AD patients and *App*^NL−F/NL−F^ knock-in mice and that the ligand bTVBT2 can effectively detect these aggregates. We also show that the density of bTVBT2-containing microglia increases alongside brain AD pathology and we propose the microglial event as an event implicated in retinal tauopathy. These findings thereby not only support previous findings demonstrating the presence of tauopathy in the AD retina, but further suggest a specific role of microglia, an event which could potentially be monitored using fluorescent ligands and imaging techniques in the future.

### Supplementary Information

**Additional file 1.**

**Additional file 2.**

## Data Availability

All data generated or analyzed during this study are included in this published article (and its supplementary information files).

## Abbreviations

AD: Alzheimer’s disease
Aβ: Amyloid beta
AppNL−F/NL−F knock-in mice: Mice expressing humanized Aβ with the Swedish and Iberian mutations
APOE genotype: Apolipoprotein E genotype
BBB: Blood-brain barrier
BRB: Blood-retina barrier
CBD: Corticobasal degeneration
FTD: Frontotemporal dementia
IAPP: Islet amyloid polypeptide
NBB: The Netherlands Brain Bank
NT: Neurophil threads
NFT: Neurofibrillary tangles
pTDP-43: Phosphorylated TAR DNA binding protein 43
p-tau: Hyperphosphorylated tau
PSP: Progressive supranuclear palsy
WT: Wild type
