# Supplementary figures and images for: The fluorescent ligand bTVBT2 reveals increased p-tau uptake by retinal microglia in Alzheimer’s disease patients and AppNL−F/NL−F mice
Source: Alzheimers Res Ther. 2024 Jan 2;16:4. doi: 10.1186/s13195-023-01375-7 (PMC10763304; doi:10.1186/s13195-023-01375-7)

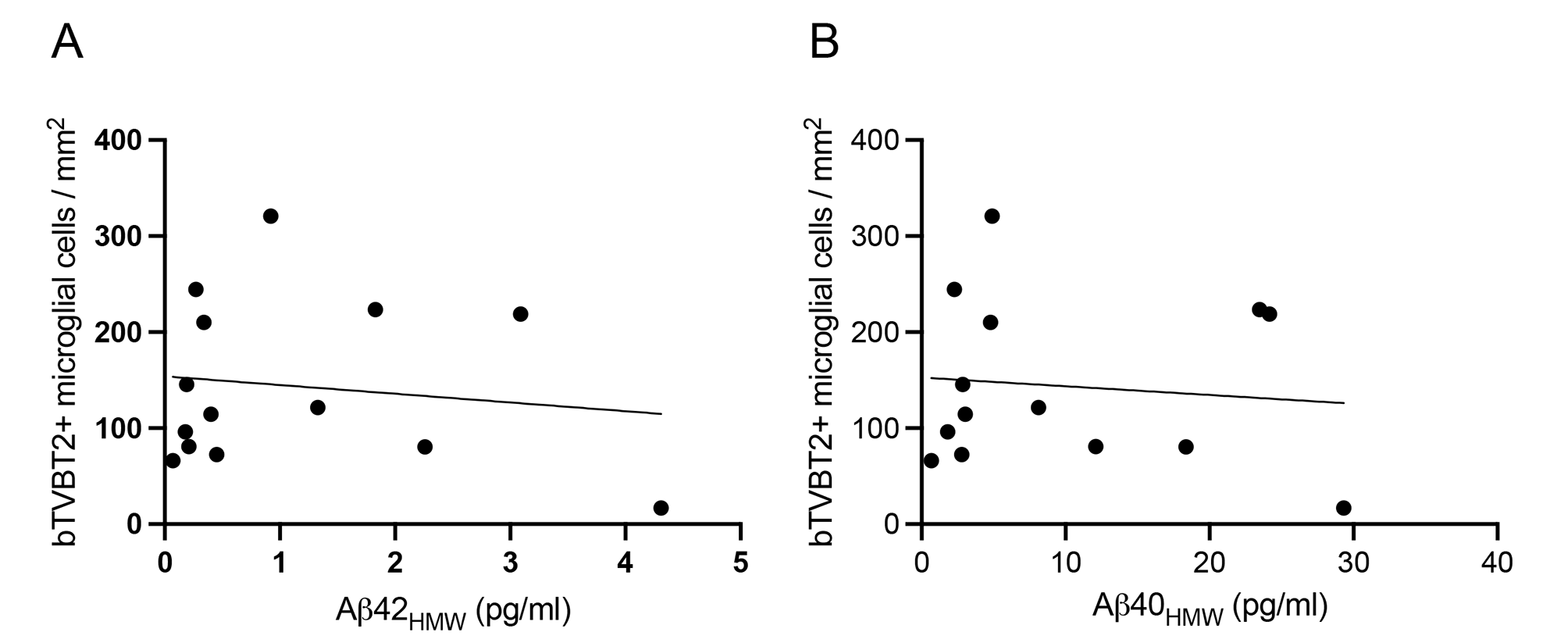

Supplement: Supplementary file 1 — Additional file 1. [file 13195_2023_1375_MOESM1_ESM.tif]

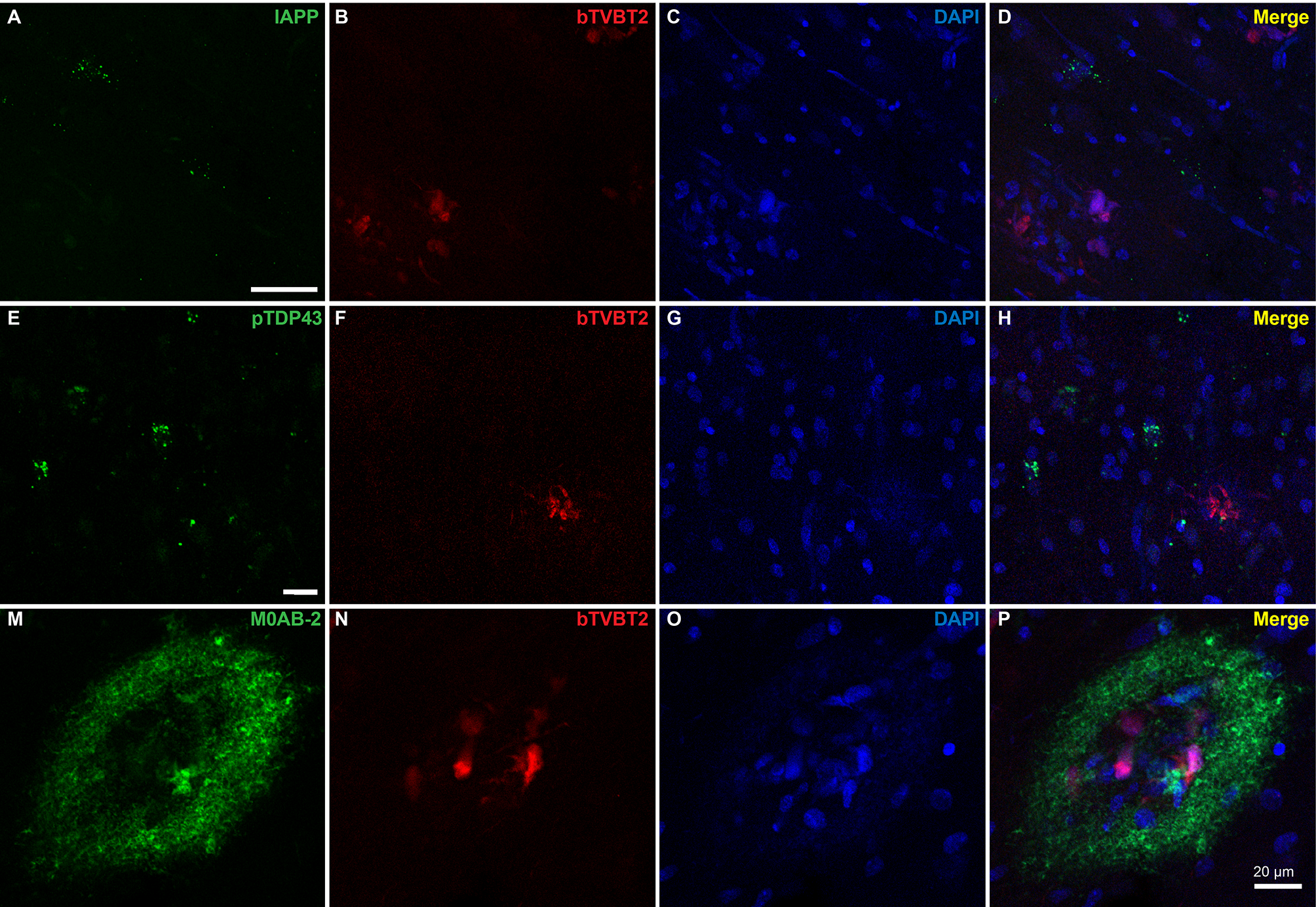

Supplement: Supplementary file 2 — Additional file 2. [file 13195_2023_1375_MOESM2_ESM.tif]
